# Inborn errors of immunity and its clinical significance in children with lymphoma in China: a single-center study

**DOI:** 10.1016/j.jped.2024.02.002

**Published:** 2024-03-25

**Authors:** Chao Yang, Nan Li, Meng Zhang, Shuang Huang, Ling Jin, Shu-Guang Liu, Chun-Ju Zhou, Zhi-Gang Li, Yan-Long Duan

**Affiliations:** a Hematologic Disease Laboratory, Hematology Center, Beijing Key Laboratory of Pediatric Hematology Oncology, National Key Discipline of Pediatrics (Capital Medical University), Key Laboratory of Major Diseases in Children, Ministry of Education, Beijing Pediatric Research Institute, Beijing Children's Hospital, Capital Medical University, National Center for Children's Health, Beijing, China; b Medical Oncology Department, Pediatric Oncology Center, Beijing Children's Hospital, Capital Medical University, National Center for Children's Health, Beijing Key Laboratory of Pediatric Hematology Oncology, National Key Clinical Discipline of Pediatric Oncology, Key Laboratory of Major Diseases in Children, Ministry of Education, Beijing, China; c Department of Pathology, Beijing Children's Hospital, Capital Medical University, National Center for Children's Health, Key Laboratory of Major Diseases in Children, Ministry of Education, Beijing, China

**Keywords:** Inborn errors of immunity, Pediatric lymphoma, Genes, Mutation, Clinical features, China

## Abstract

**Objective:**

To investigate the incidence, clinical and genetic characteristics of pediatric lymphoma patients of China with inborn errors of immunity (IEI)-related gene mutations, which have not been fully studied.

**Method:**

From Jan. 2020 to Mar. 2023, IEI-related genetic mutations were retrospectively explored in 108 children with lymphomas admitted to Beijing Children's Hospital by NGS. Genetic rule and clinical characteristics as well as treatment outcomes were compared between patients with or without IEI-related gene mutations.

**Results:**

A total of 17 patients (15.7 %) harbored IEI-associated mutations, including 4 cases with X-linked lymphoproliferative syndrome (XLP), 3 cases had mutations in tumor necrosis factor receptor superfamily 13B (TNFRSF13B), 2 cases with Activated p110 syndrome (APDS). Patients with IEI all had alteration of immunocompetence with decreased levels of immunoglobulin and lymphocyte subsets. Recurrent infection existed in 41.2 % of patients. The 18-month event-free survival (EFS) and the overall response rate (ORR) of patients with IEI are significantly lower than those without IEI (33.86% vs. 73.26 %, *p* = 0.011; 52.94% vs. 87.91 %, *p* = 0.002, respectively). In addition, patients with IEI had a higher progression disease (PD) rate of 23.5 % than those without IEI of 4.4 % (*p* = 0.006).

**Conclusion:**

The present study demonstrated that IEI-associated lymphomas were much more common than originally appreciated in pediatric lymphomas, and those were insensitive to treatment and more likely to progress or relapse. The genomic analysis and a thorough review of the medical history of IEI can be used to distinguish them from pediatric lymphomas without IEI, which are beneficial for the early diagnosis and direct intervention.

## Introduction

Leukaemias, CNS tumors, and lymphomas are the pediatric cancers with the highest incidences in China. Among them, lymphoma (11.54 per million) accounts for 9.7 % of pediatric cancers, which may be an underestimated number.^1^^,^^2^ Pediatric and teenage lymphoma is characterized by a high degree of malignancy and aggressiveness.^3^ Based on the latest WHO classification (version 2022), lymphoma is divided into precursor cell neoplasms and mature malignancies of different cell lineages.^4^ Lymphoma in children and adolescents could be caused by infection, genetic, immunological, and physicochemical factors. At present, pediatric and adolescent lymphoma patients are treated with chemotherapy, radiotherapy, stem cell transplantation, and so on. In addition, immunotherapy has further improved the treatment outcome and prolonged the survival time of patients.^5^^,^^6^

Recently, new understandings of lymphoproliferative disorders associated with inborn errors of immunity (IEI, previously known as primary immunodeficiencies) and acquired immune disorders have been developed and updated in WHO-HAEM5.^4^ IEI comprise at least 450 inherited diseases. Arisen from intrinsic defects in immunity mostly due to genetic mutations, IEI patients are prone to recurrent infections, autoimmunity, inflammation, allergy, and malignancy.^7^ Lymphoma is the main cancer type in patients with IEI, especially in children.^8^^,^^9^ Molecular signaling abnormalities, defective immune surveillance, persistent stimulation by pathogens or transforming viruses, and other unknown mechanisms lead to an enhanced risk of lymphoma in patients with IEI.^10^^,^^11^ On the other hand, lymphoma can also reveal an underlying IEI. For example, lymphoma develops before the onset of PID in 11 % of cases.^12^

Lymphoid malignancies in patients with IEI are clinically and histologically heterogeneous, hampering prompt and accurate diagnosis of IEI.^13^^,^^14^ In the last decade, advances in gene sequencing technologies have facilitated the diagnosis of IEI-associated lymphoma. Pediatric lymphoma patients with heterogeneous genetic defects are much more common than previously considered.^11^^,^^15^^,^^16^

To date, the correlation between genetic abnormalities and clinical features in pediatric patients with IEI-associated lymphoma has not been fully investigated, especially in China. In this study, the authors retrospectively analyzed the clinical features and treatment outcomes in 17 pediatric patients with IEI-associated lymphoma.

## Materials and methods

### Patients

One hundred and eight patients with lymphoma treated at the Hematology Oncology Center of Beijing Children's Hospital were retrospectively enrolled in this study from Jan. 2020 to Mar. 2023. The exclusion criterion included children unable to regularly receive and complete chemotherapy or those who refuse to do tests by NGS for other reasons. Lymphomas were diagnosed by more than two pathologists, as collected and summarized their clinical and laboratory characteristics in Table S1. An evaluation system was developed and adopted for patients who were detected with genetic variations related to IEI. The evaluation system was devised as follows:

1) IEI-group: Patients with lymphoma who had positive IEI-related mutations fulfilled the following criteria: (a)–(b).
2) Non-IEI group: Patients with lymphoma who did not have positive IEI-related mutations.
  (a) Positive IEI-relevant phenotype (characterized by autoimmunity, allergic disease, and/or immune deficiency) or positive family history of autoimmunity, allergic disease, and/or recurrent infections.
  (b) Decreased concentrations of Igs (a marked decrease in the levels of at least one of the isotypes IgG, IgM, or IgA) or lymphocyte subsets(at least one of the absolute numbers of B cells, T cells, or NK cells).

### Samples

DNA was extracted from the peripheral blood of the patients at diagnosis and their parents (when possible) using the QIAamp DNA Mini Kit (Qiagen, Shanghai, China) using the manufacturer's instructions. Clinical data including demographic characteristics, family history, past medical history, treatment outcomes, and clinical laboratory tests were collected, as summarized in Table S1. Informed consent for the study, including consent for the collection and the use of DNA samples for genetic analysis, was obtained from eligible children and their parents or legal guardians. The study for genetic analysis was approved under the guidelines of the ethics committee of Beijing Children's Hospital (China).

### Targeted sequencing

To gain an insight into the potential lymphoma-associated IEI genes/targets, the authors designed a sequencing panel containing 152 IEI genes associated with malignancies (Table S2) based on the previous reports on lymphoma or pan-cancer IEI studies.^7^^,^^10^ Extracted DNA was sent to MyGenostics Company for targeted sequencing using a gene capture strategy with a GenCap custom enrichment kit (MyGenostics, China) following the manufacturer's protocol. Nucleotide-targeted sequencing was performed on the Illumina HiSeq 2000 platform (Illumina, USA). The mutations in IEI-related genes and other lymphoma susceptibility and related genes were verified by Sanger sequencing in the patients and their parents (when possible).

### Mutational signature analysis

After sequencing, the raw data was saved in FASTQ file format. Illumina sequencing adapters and low-quality reads (<80 bp) were screened out using Cutadapt. The SIFT database and polymorphism phenotyping v2 were used to predict the pathogenicity of single nucleotide polymorphisms.^17^^,^^18^ In order to verify the pathogenic mutations, the authors collected data from the Clinvar database (https://www.ncbi.nlm.nih.gov/clinvar/) or the American College of Medical Genetics and Genomics (ACMG) guidelines.

### Statistics

SPSS version 20.0 (IBM, Armonk, USA) was used for all statistical analyses of the data. The *t*-test and chi-square test were used to determine the differences between two numerical variables obeying normal or skewed distribution, respectively. Pearson's chi-square test was used to determine whether there was a difference in qualitative variables. Events were defined as follows: disease progression, relapse, or death for any reason. EFS was estimated by Kaplan-Meier survival analysis and compared with the log-rank test. A p value < 0.05 was considered to be statistically significant.

## Results

### General information

There were 27 (25 %), 38 (35 %), 18 (16 %), and 25 (23 %) patients with precursor lymphoid neoplasms, Mature B-cell neoplasms, Mature T- and NK-cell neoplasms, and HL, respectively. Among them, 24 cases were infected with EBV. There were 87 boys and 21 girls with a sex ratio of 4.35 to 1. The median age was 8.48 (range: 1–17.58) years at diagnosis (Table S1).

### Mutations in IEI-related genes in children with lymphoma

All 108 children carried genes associated with immunodeficiency. The authors identified 17 pathogenic or likely pathogenic variants by SIFT, Polyphen2 software, clinvar database, and the (ACMG) guidelines. The overall diagnostic yield of 108 patients was 15.7 % (17/108). Mutations in *SH2D1A, NFKB2, STAT1, PALB2, TNFRSF13B, CTLA4, PIK3CD, ELANE, POLE, MSH6, MCM4* gene were detected (Table 1).

**Table 1:** Genetic results for 17 lymphoma patients with mutations in IEI-associated gene.

Patient ID	Diagnosis	Gene	Genomic variant(s)	Reference sequence	Zygosity	Inherited pattern	SIFT	PP2	MT	GERP++	ACMG scoring	ACMG pathogenicity	Source of variation	Type of mutation
PL 1	XLP1	*SH2D1A*	c.145G>*A*/p.G49S	NM_002351	hemi	XL	T	B	D	C	PM1;PM2	UC	Mo	nonsynonymous
PL 2	XLP1	*SH2D1A*	exon2 deletion	NM_002352	hemi	XL	-	–	–	–	PVS1+PS4+PM2	P	–	
PL 3	XLP1	*SH2D1A*	c.166dupG/p.V56Gfs*12	NM_002351	hemi	XL	-	–	–	–	PVS1;PM1;PM2	P	Mo	frameshift
PL 4	XLP1	*SH2D1A*	c.164G>*A*/p.R55Q	NM_002351	hemi	XL	D	PD	D	C	PS1;PM1;PM2;PP3	LP	SP	nonsynonymous
PL 5	CVID	*NFKB2*	c.1347delC/p.L450Cfs*32	NM_001077494	het	AD	-	–	–	–	PVS1;PM2	LP		frameshift
PL 8	CVID	*STAT1*	c.778_779insC/p.Q260Pfs*24	NM_007315	Compound het	AR	-	–	–	–	PVS1;PM2	LP		frameshift
		*STAT1*	c.770delA/p.D257Vfs*9				-	–	–	–	PVS1;PM2	LP		frameshift
PL 22	CVID	*PALB2*	c.2748+1G>*A*/splicing	NM_024675	het	AD	-	–	D	C	PVS1;PS1;PM1;PM2	P		splicing
PL 37	CVID	*TNFRSF13B*	c.105delC/p.E36Kfs*48	NM_012452	het	AD	-	–	–	–	PVS1;PS1;PM2	P		frameshift
PL 39	CVID	*CTLA4*	c.163_164insCC/p.S55Tfs*20	NM_005214	het	AD	-	–	–	–	PVS1;PM1;PM2	P		frameshift
PL 42	APDS	*PIK3CD*	c.2126C>A/p.P709H	NM_005026	het	AD	T	PD	D	C	PM2	UC	Fa	nonsynonymous
PL 43	APDS	*PIC3CD*	c.3061G>*A*/p.E1021K	NM_005026	het	AD	D	PD	D	C	PS1;PM2;PP3	LP	Mo	nonsynonymous
PL 57	CVID	*TNFRSF13B*	c.226G>*A*/p.G76S	NM_012452	het	AD	D	PD	D	C	PM1;PM2;PM5;PP3	LP		nonsynonymous
PL 58	CVID	*ELANE*	c.547_548insAC/p.R183Hfs*11	NM_001972	het	AD	-	–	–	–	PVS1;PM1;PM2	P		frameshift
PL 62	CID	*POLE*	c.6532–5C>T/splicing	NM_006231	Compound het	AR	-	–	–	–	PM2	UC		splicing
		*POLE*	c.6401_6402insGC/p.D2134Efs*99				-	–	–	–	PVS1;PM2	LP		frameshift
PL 84	CVID	*TNFRSF13B*	c.572dupA/p.D191Efs*46	NM_012452	het	AD	-	–	–	–	PVS1;PS1;PM1;PM2	P	Mo	frameshift
PL 87	CVID	*MSH6*	c.2561A>*T*/p.K854M	NM_000179	het	AD	D	PD	D	C	PM2;PP3	US		nonsynonymous
PL 108	CID	*MCM4*	c.2572delA/p.K858Rfs*10	NM_005914	Compound het	AR	-	–	–	–	PVS1;PM2	LP		frameshift
		*MCM4*	c.2582delG/p.R861Pfs*7				-	–	–	–	PVS1;PM2	LP		frameshift

CID, Combined Immune Deficiency; APDS, Activated p110δ syndrome; CVID, Common variable immunodeficiency; XLP1, X-linked lymphoproliferative disease; PP2, polymorphism phenotyping v2 (PD, possible damaging; B, benign); SIFT, sorting intolerant from tolerant (D, damaging; T, tolerant); clinvar (P, pathogenic; LP, likely pathogenic; CP, Conflicting interpretations of pathogenicity; US, uncertain significance); ACMG, American college of medical genetics and genomics (P, pathogenic; LP, likely pathogenic; UC, uncertain). Fa, father; Mo, mother; SP, spontaneous.

According to the 2022 update of IUIS phenotypical classification for human inborn errors of immunity,^7^ these patients included 4 cases with X-linked lymphoproliferative syndrome (XLPS) (4/17, 23.5 %), 8 of whom were diagnosed with common variable immunodeficiency (CVID) (8/17, 47.1 %), 2 with activated phosphoinositide 3-kinase delta (PI3Kδ) syndrome (APDS), and an additional 2 patients with combined immunodeficiency (CID) (2/17, 11.8 %). Autosomal dominant (AD), autosomal recessive (AR), and X-linked recessive (XLR) diseases were observed in 10 (58.8 %), 3 (17.6 %), and 4 (23.5 %) patients, respectively. Hemizygous pathogenic mutations were all in *SH2D1A* of 4 XLP patients, and heterozygous pathogenic mutations were found in the rest of 13 patients. Among 17 positive IEI-related mutations, only six patients and their parents were verified by sanger sequencing (Supplementary Figure 1). Of the 4 XLR cases, 1 variant was de novo of patient 4. Patients 1 and 3 inherited from their mothers, respectively. Reviewing the family history of patient 3, the authors found the mother had recurrent respiratory infections during childhood. Hemizygote deletion of exon 2 had been detected in *SH2D1A* of patient 2, yet no pedigree validation was conducted. P.P709H and p.E1021K mutations of *PIK3CD* were found in patients 42 and 43 inherited from her father and mother respectively, who had suspicious clinical manifestations related to IEI. Patient 84 had a mutation of *TNFRSF13B* inherited from his mother, who was diagnosed with inflammatory bowel disease(IBD) (Table 2 and Table S2). The type of mutation that causes the most immune deficiency mutations in patients was frameshift (8/17, 47.06 %), followed by nonsynonymous mutation (6/17, 35.3 %) and 2 splicing mutations (Table 1).

**Table 2:** Comparison of clinical characteristics in pediatric lymphoma patients with or without IEI in this study.

Clinical characteristics	Total	IEI	Non-IEI	*P*-value
N	108	17	91	
Sex				
Male	87(80.6 %)	15(88.4 %)	72(79.1 %)	0.760
Female	21(19.4 %)	3(11.6 %)	19(20.9 %)	
Median age (range), y	7.41(0.5–17.9)	8.75(0.5–17.9)	7.37(1–17.9)	0.218
Stage				
Ⅰ/Ⅱ	25(23.1 %)	**4(**23.5 %)	21(23.1 %)	0.221
Ⅲ/Ⅳ	83(76.9 %)	13(76.5 %)	70(76.9 %)	
EBV	23(21.3 %)	3(17.6 %)	20(22 %)	0.689
Pathological types				
Precursor lymphoid neoplasms	27(25 %)	4(23.5 %)	23(25.3 %)	0.879
Mature B-cell neoplasms	38(35.2 %)	7(41.2 %)	31(34.1 %)	0.537
Mature T- and NK-cell neoplasms	18(16.7 %)	3(17.6 %)	15(16.5 %)	0.906
Hodgkin lymphomas	25(23.1 %)	3(17.6 %)	22(24.2 %)	0.558
Past history related to immune defects	18	10(58.8 %)	11(12.1 %)	0.000⁎⁎
Family history related to immune defects	8	8(47.1 %)	0	0.000⁎⁎

⁎⁎ Indicates a p-value of < 0.01.

### Clinical characteristics of IEI related lymphoma patients

To determine whether the genotype of patients with IEI is associated with the lymphoma phenotype, the authors further compared the clinical characteristics and laboratory tests of patients in this cohort. There were 14 (88.4 %) boys and 3 (11.6 %) girls with IEI. The median age was 8.75 years (range, 0.5–17.91). 4 patients were diagnosed with precursor lymphoid neoplasms. The remaining 13 patients developed DLBCL (*n* = 4), BL (*n* = 2), HGBL (*n* = 1), ALCL (*n* = 3), and HL (*n* = 3). Among the disease stages, stage II (*n* = 4, 23.5 %), stage III (*n* = 7, 41.2 %), stage IV (*n* = 6, 35.3 %). Comparing clinical characteristics between pediatric lymphoma patients with or without IEI, pathological types, and the stage, showed no significant difference between the two groups and no correlation with IEI (all p>0.05) (Tables 2 and 3).

**Table 3:** Clinical features in 17 lymphoma patients with IEI-associated mutations.

Patient ID	Gene	Lymphoma	Sex	Age	Stage	Infection	Decreased Igs(g/L)	B-cell(%)	T-cell(%)	NK-cell(%)	Indicators of immune dysregulation	Family history related to IEI
PL 1	*SH2D1A*	BL	Male	12.16	Ⅱ	RRI	IgG(2.12)	Low(3.3)	–	–	Recurrent eczema	–
PL 2	*SH2D1A*	HGBL	Male	5.33	III	–	IgM(0.21)	Low(12..5)	–	–	–	–
PL 3	*SH2D1A*	DLBCL	Male	17.91	III	–	–	Low(9.5)	Low(35.7)	–	Allergy	Fa:+
PL 4	*SH2D1A*	DLBCL	Male	4.66	III	RRI;TB infection	IgG(4.09)	–	–	Low(6)	–	
PL 5	*NFKB2*	DLBCL	Male	11.5	IV	EBV and HP infection	–	Low(0.6)	–	Low(2.8)	–	
PL 8	*STAT1*	MCCHL	Male	5	II	EBV infection	IgM(0.36)	Low(12.5)	–	–	Recurrent eczema	Mo:+
PL 22	*PALB2*	ALK+-ALCL	Male	12	IV	–	IgG(4.21); IgM(0.11)	Low(10.8)	–	Low(3.1)	–	–
PL 37	*TNFRSF13B*	MCCHL	Male	8.75	II	EBV infection	IgM(0.38)	Low(4.3)	Low(27.8)	Low(6.9)	–	Mo:+
PL 39	*CTLA4*	T-LBL	Male	7.58	IV	RRI	–	Low(10.5)	Low(30.3)	Low(3.2)	–	Fa:+:
PL 42	*PIK3CD*	ALK+-ALCL	Female	17.9	III	–	IgM(0.37)	Low(10.2)	–	–	HLH	–
PL 43	*PIK3CD*	B-LBL	Female	5	III	RRI	IgM(0.26)	Low(6.7)	Low(37.5)	–	ANAs+	
PL 57	*TNFRSF13B*	B-LBL	Female	2.5	IV	–	IgA(0.12)	–	–	Low(4.4)	Recurrent eczema	Mo:+
PL 58	*ELANE*	BL	Male	14.16	III	HP infection	IgM(0.63)	Low(5.7)	–	–	–	Mo:+
PL 62	*POLE*	NSCHL	Male	0.5	II	RRI	–	Low(7.5)	–	Low(4.4)	–	–
PL 84	*TNFRSF13B*	B-LBL	Male	5.5	III	–	IgM(0.26)	Low(12.5)	Low(24.2)	–	Allergy urticaria	Mo:+
PL 87	*MSH6*	ALK+-ALCL	Male	9.08	IV	RRI; bronchopneumonia	–	–	Low(23)	Low(3)	HLH	–
PL 108	*MCM4*	DLBCL	Male	12	IV	RRI;bronchopneumonia	IgG(3.59)	Low(10)	–	Low (2.1)	Allergy	–

BL, Burkitt's lymphoma; HGBL, high-grade B lymphoma; DLBCL, diffuse large B-cell lymphoma; MCCHL, mixed cellularity classic Hodgkin lymphoma; NSCHL, nodular sclerosis Hodgkin's lymphoma; ALK+-ALCL, ALK positive anaplastic large cell lymphoma; T-LBL, T-lymphoblastic lymphoma; B-LBL, B-lymphoblastic lymphoma; RRI, recurrent respiratory infections; HLH, hemophagocytic lymphohistiocytosis; HP, helicobacter pylori; Fa, father; Mo, mother.

Regarding infections, 3 (17.6 %) patients had EBV infection, one of them had a combined HP infection. In addition, one case of tubercle bacillus infection was found in the rest people. 2(11.8 %) patients had hemophagocytic lymphohistiocytosis during the treatment. For the non-IEI group, 22 % of patients had EBV infection, which showed no significant difference between the two groups.

Regarding past history, 58.8 % (10/17) of patients in the IEI group had a positive past history of recurrent upper respiratory tract infections, eczema or allergy; and 47.1 %(8/17) of patients’ parents had positive family history of recurrent respiratory tract infections and allergic diseases, including cancer, allergic asthma, and allergic rhinitis. However, in the non-IEI group, only 8.5 % of patients had a related positive phenotype. None of the patients in the non-IEI group had a related family history. The differences between the two groups were statistically significant.

The level of immunoglobulin and immunophenotyping of peripheral blood lymphocyte subsets can provide important information for the diagnosis and treatment of immunological and hematological disorders.^19^ Prominently, the incidence of hypogammaglobulinemia or the decline of immunophenotyping of lymphocyte subsets were 100 % in IEI-group. For those in the non-IEI group, 5.5 % had hypogammaglobulinemia or a low level of immunophenotyping of lymphocyte subsets.

### Correlation of the mutations in IEI-related genes with treatment response and prognosis

**Figure 1:**
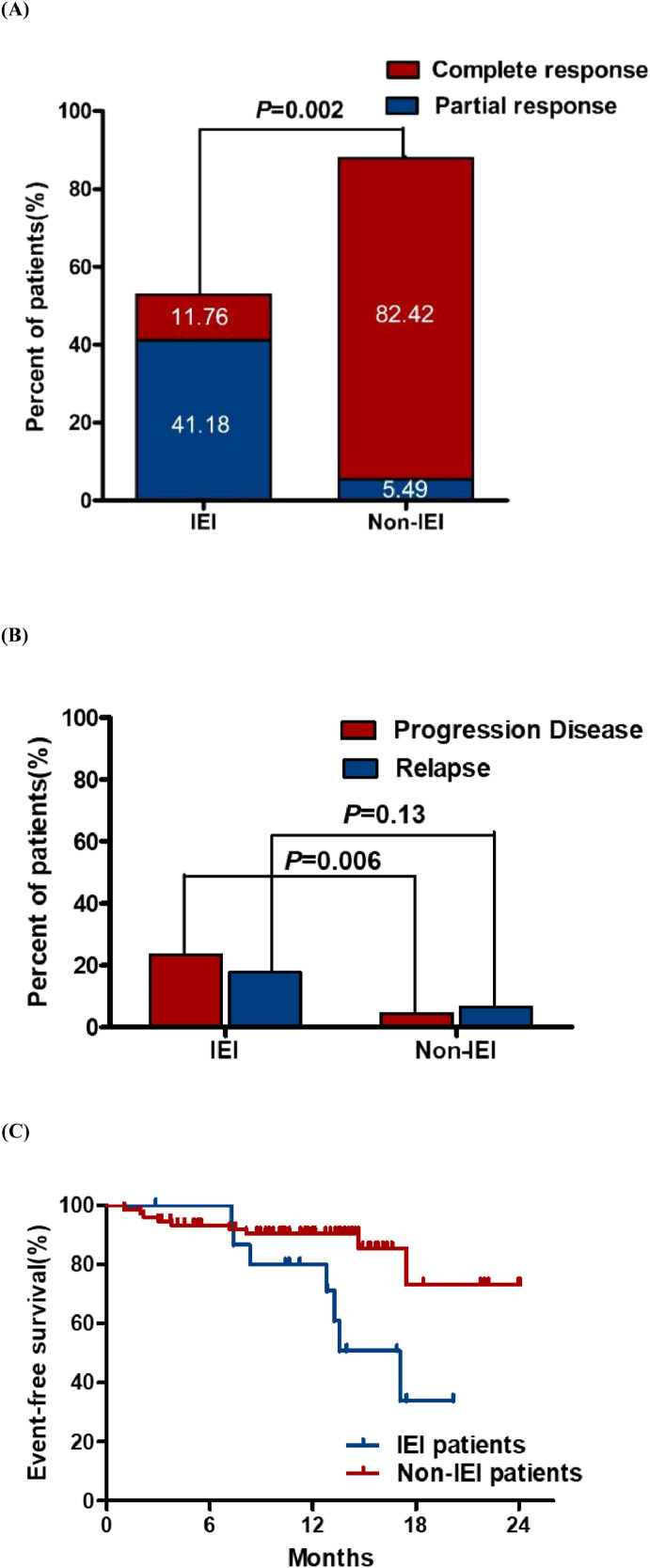
Comparison of response outcomes for lymphoma patients with or without IEI. (A). Overall response (ORR) and (B) the rate of progressive/relapse at the end of treatment. CR, complete response; PR, partial response. (C) Eighteen-month Event-free survival rates of lymphoma patients. EFS of patients was 33.9 % [95 % CI, 31.3 to 36.5] for the IEI group and 73.2 % [95 % CI, 71.09–75.31] for the non-IEI group.

Two (11.76 %) of 17 patients with IEI achieved complete response (CR) at the end of treatment overall, and 7(41.18 %) patients achieved partial response (PR). The overall response rate (ORR) at the end of therapy exhibited a significant difference between the patients with or without IEI (52.94% vs. 87.91 %, *p* = 0.002, Figure 1A).

Notably, there were 7 patients with IEI experienced events, including 4 (23.53 %) patients with progressive disease (PD) and 3 (17.65 %) patients with disease relapses. The PD rate of patients with IEI was higher than those without IEI by 4.40 % (*p* = 0.006, Figure 1B). Among the patients who progressed during treatment, 2 patients were given salvage chemotherapy, one received rituximab combined chemotherapy, and another had chemotherapy and allogeneic hematopoietic stem cell transplantation(allo-HSCT). The sites of relapse in the patients were CNS and the primary tumor. Patients who progress or relapse achieve only partial remission after treatment, which only achieved PR after treatment. All patients who achieved PD or relapse only maintained PR throughout the whole follow-up period. The 18-month EFS of patients with IEI was lower than those without IEI (33.9 % [95 % CI, 31.3 to 36.5] vs. 73.2 % [95 % CI, 71.09–75.31], *p* = 0.011, Figure 1C), indicating that the IEI status is significantly associated with treatment response and prognosis.

## Discussion

Inborn errors of immunity (IEI), with most due to genetic mutations, and comprise over 485 diseases that could present with a diverse range of disorders including infection, autoimmunity, inflammation, malignancy, and allergy.^7^ The incidence of IEI in international registries is 11.2 per 100,000 births,^19^^,^^20^^,^^21^ and the risk of developing cancer in children with IEI is about 5 to 25 %.^22^ However, lymphoma is the most common type of tumor. The largest study of 3658 patients with IEI registered in the United States Immune Deficiency Network (USIDNET) reported 48 % lymphoma cases.^19^ Based on the European Society for Immunodeficiencies (ESID) database, 8.1 % of Dutch patients with IEI had malignancy, of which lymphoma was the most frequent cancer (28.3 %) with NHL.^23^ Immunodeficiency is more common in lymphoma than the authors thought, especially in children and adolescents. In addition, many overlapping phenotypes of lymphoma and IEI, and all these diseases could manifest with a wide range of phenotypes of varying severities, resulting in difficult diagnosis of IEI timely and accurately, especially in resource-limited countries and regions.^24^ In China, there are no large cohort studies and surveys, especially in pediatric lymphomas. Therefore, the study of IEI-associated lymphomas has important clinical significance.

This study retrospectively analyzed IEI-associated mutations, clinical characteristics, and treatment outcomes of pediatric lymphoma patients with IEI. Pediatric IEI-associated lymphoma showed particular genetic background, and clinical and biological features at diagnosis, compared with patients without IEI.

The data showed that IEI occurred in 15.7 % (17/108) of the Chinese pediatric lymphoma cohort, which approached to that 17 % (17/100) of Turkish pediatric oncology centers.^25^ Of the positive cases, the most frequent IEI type were CVID and XLP1. IEI diseases are genetically and phenotypically heterogeneous disorders,^8^ the data of mutations indicates similar results. Among IEI-associated genes,^7^^,^^9^ *NFKB2*,^26^ *PIK3CD, STAT1*,^27^ *PALB2, SH2D1A*,^28^ *TNFRSF13B*,^10^ *MSH6, CTLA4* genes had previously been reported in lymphoma or hematological malignancies genomic studies or are lymphoma susceptibility genes. By sanger sequencing, 66.7 % (6/9) of children were inherited from parents carriers. Therefore, necessary fertility guidance of immunodeficiency gene screening is helpful for those families.

In IEI cases, the risk of developing lymphoma, particularly NHL, with about 40–50 % of those diagnosed with DLBCL.^6^^,^^11^ In this study, a diagnosis of IEI was made in 82.4 % (14/17) children of NHL, especially mature b-cell neoplasms (41.2%). In addition, EBV infections are known to be related to lymphomagenesis in patients with IEI.10,36 There were only 3 patients in the IEI group who were EBV+, lower than the non-IEI group. Pan-Hammarström et al. reported that a different mutational profile may exist in the EBV+ lymphoma genome from that in IEI patients.^10^

Among the many types of IEI, genetic defects could cause abnormalities in the differentiation and development of T and B cells.^7^ Abnormalities occurring at different stages often lead to non-specific clinical manifestations represented by recurrent/specific infections. The authors found that patients with IEI had apparent alteration of immunocompetence of low immunoglobulin or decreased T, B, and NK lymphocyte subsets, which might help to identify IEI patients in pediatric lymphomas. Most patients were observed to have a previous history of recurrent respiratory infections, eczema, and changes in allergy. Therefore, initial screening of IEI in lymphoma can be determined by laboratory tests that are less technically demanding and more available, such as complete blood count, lymphocyte subsets, immunoglobulin profile, in addition to family surveys.^29^

Despite the high rate of cure in lymphoma, disease progression/relapse occurred in cases with lymphoma developing on a background of IEI considerably increased. The data also revealed that the rate of EFS was significantly lower in the IEI group than in the group without IEI. The most important problem is the high occurrence rate of treatment-related toxicity, like mucositis, infection, and bone marrow suppression. As a result, the tumors of these patients had become refractory with reduced dosages of chemoradiotherapy, which affected the treatment outcome. So far the team has reported the treatment of Hodgkin's lymphoma (HL) in pediatric patients with titin (TTN) gene mutation and heart failure. In this case, the authors explored to use brentuximab vedotin (BV) plus chemotherapy without anthracyclines to treat one pediatric HL patient with TTN mutation. At the end of 4 cycles of BV and six courses of chemotherapy, with complete remission achieved, the tumor was reduced by 85 %.^30^ Thus, hypotoxicity and more effective targeted immunotherapy may improve the treatment success.

NGS was used to sequence highly suspected lymphoma patients, which was cost-effective and time-efficient.^13^ Notably, given the economic considerations of some families, genetic data were acquired from only 152 IEI genes associated with tumorigenesis in this study, which probably caused most of the information on the whole exome and genome to be overlooked. Whereas a public interest project from 2001 by Hong Kong University pointed to targeted gene sequencing should remain the first-tier genetic test for children to suspect common IEI.^20^ Therefore, expanding gene panel or selecting WES/WGS is a question worth considering, especially the patients in economically underdeveloped areas.

In conclusion, pediatric oncologists should be aware of the increasing trend of lymphoma patients with IEI and make early identification and diagnosis of IEI patients through clinical symptoms, family history, and immune-related laboratory tests. For diagnosed patients, adjusting treatment regimens timely, and targeted immunotherapy may be the new directions in the future. Therefore, pediatricians are required to master certain knowledge of immunology and genetics to carry out correct genetic counseling for children undergoing genetic testing, including follow-up birth planning and sibling screening. Exploring the genetic characteristics further and achieving sustained resolution of IEI-associated lymphoma remains a challenge, and further prospective studies with larger sample sizes and extended follow-up will be required.

## Conflicts of interest

The authors declare no conflicts of interest.

## Appendix Supplementary materials

Image, application 1

Image, application 2

Image, application 3
