# Supplementary material for: Inborn errors of immunity and its clinical significance in children with lymphoma in China: a single-center study
Source: J Pediatr (Rio J). 2024 Mar 25;100(4):384–91. doi: 10.1016/j.jped.2024.02.002 (PMC11331237; doi:10.1016/j.jped.2024.02.002)

**JPED-D-24-00002 – Supplementary Material**

**Figure legends**

**Supplementary Figure.1** Identified disease-causing or associated mutations of some patients(PL1, 3, 4, 42, 42, 84) and their parents were validated by Sanger sequencing.


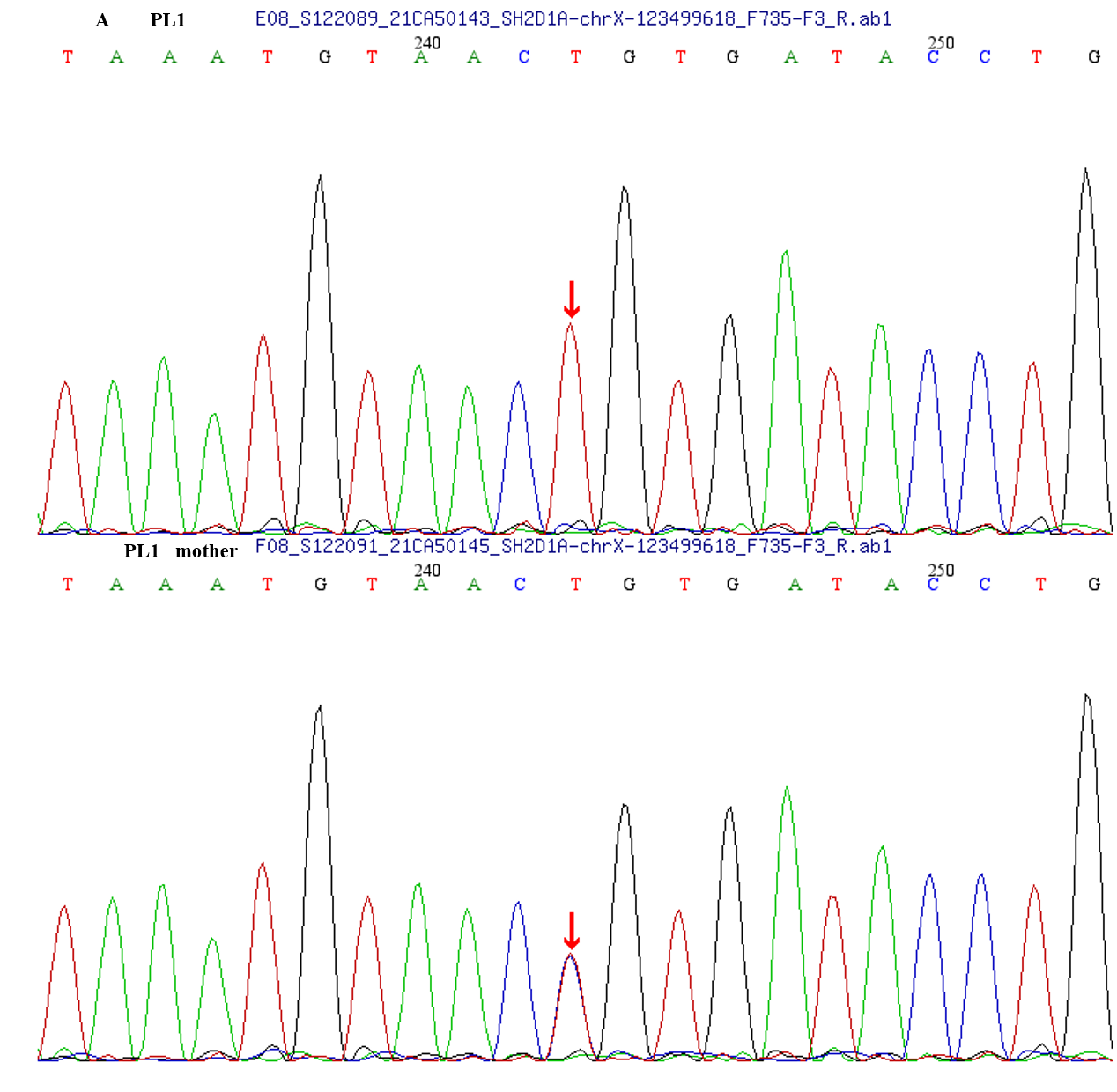


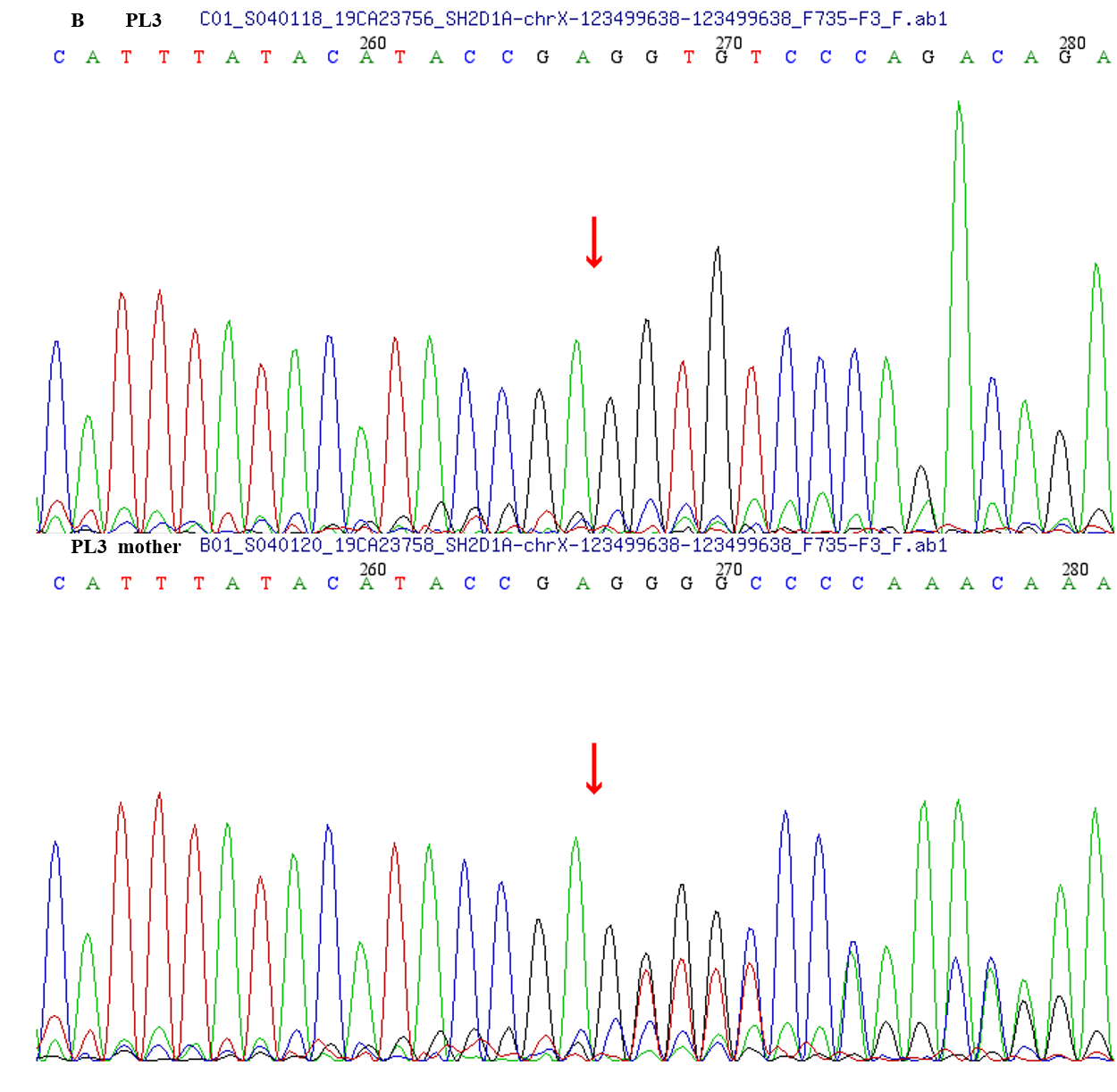

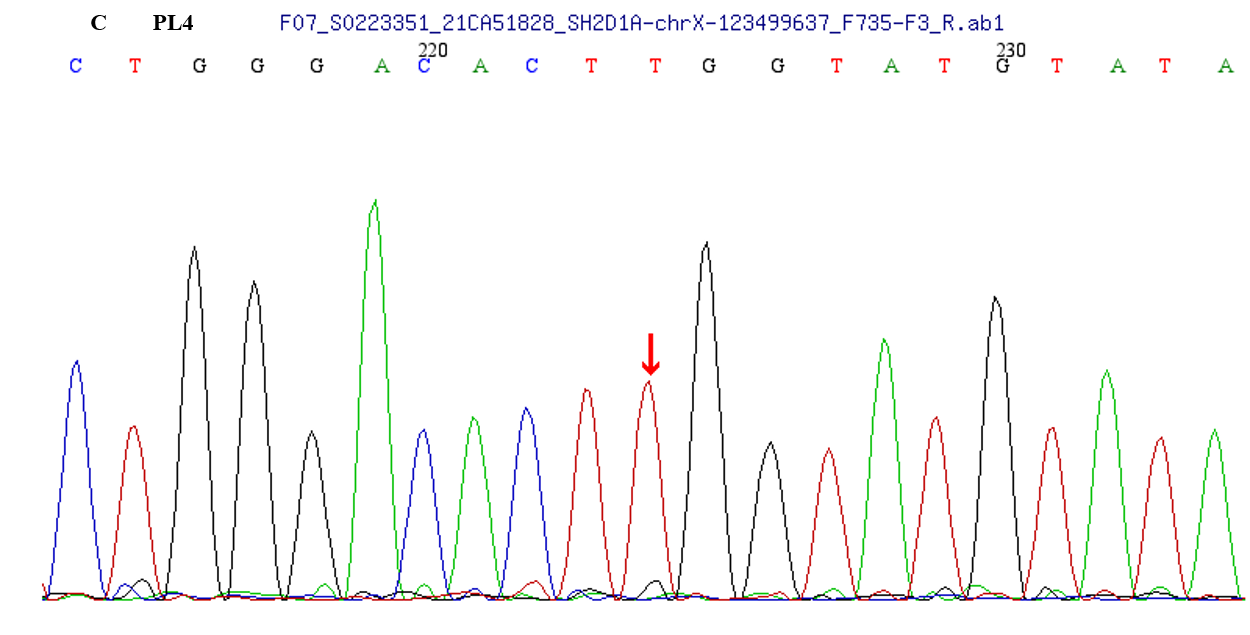

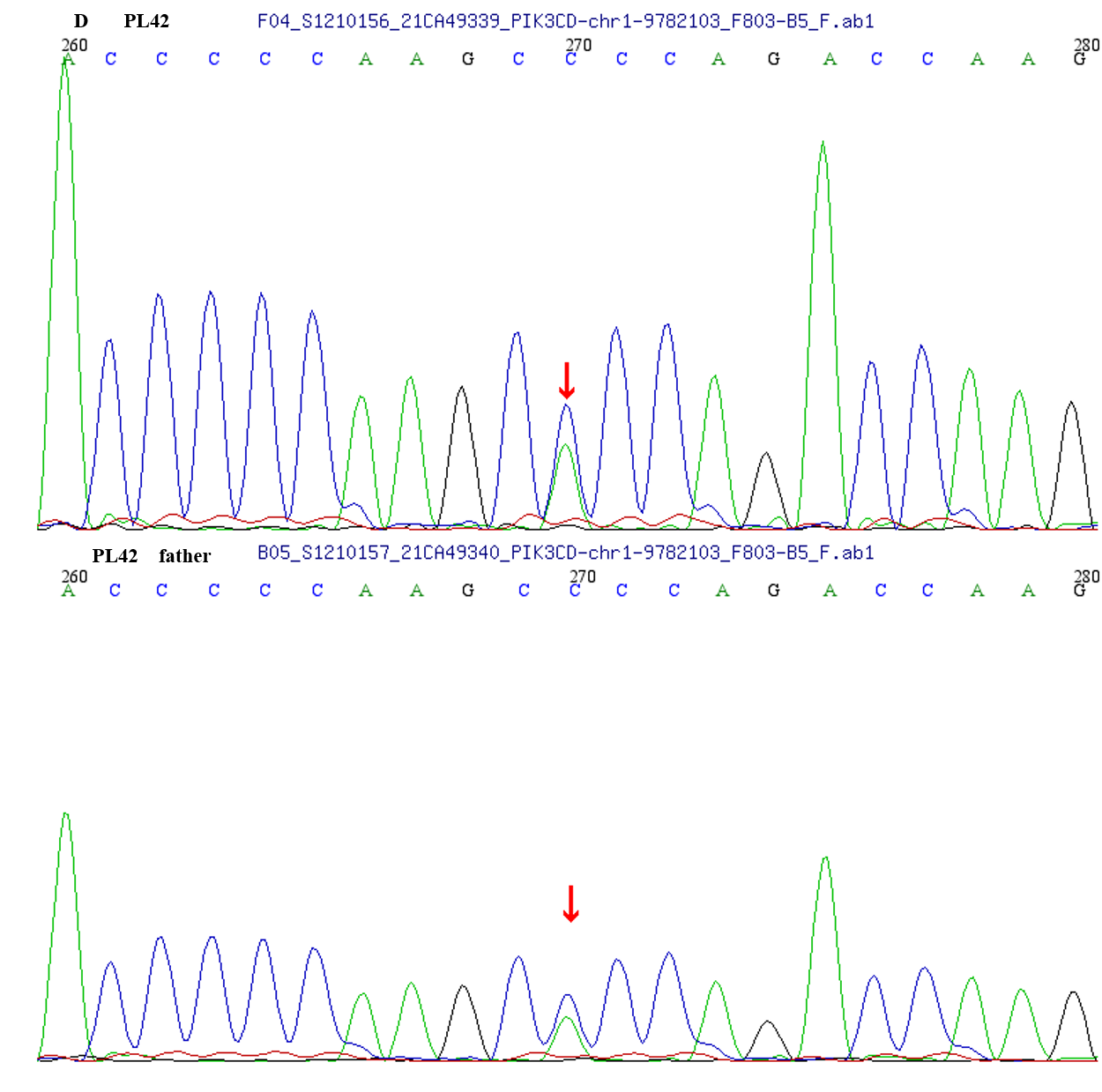

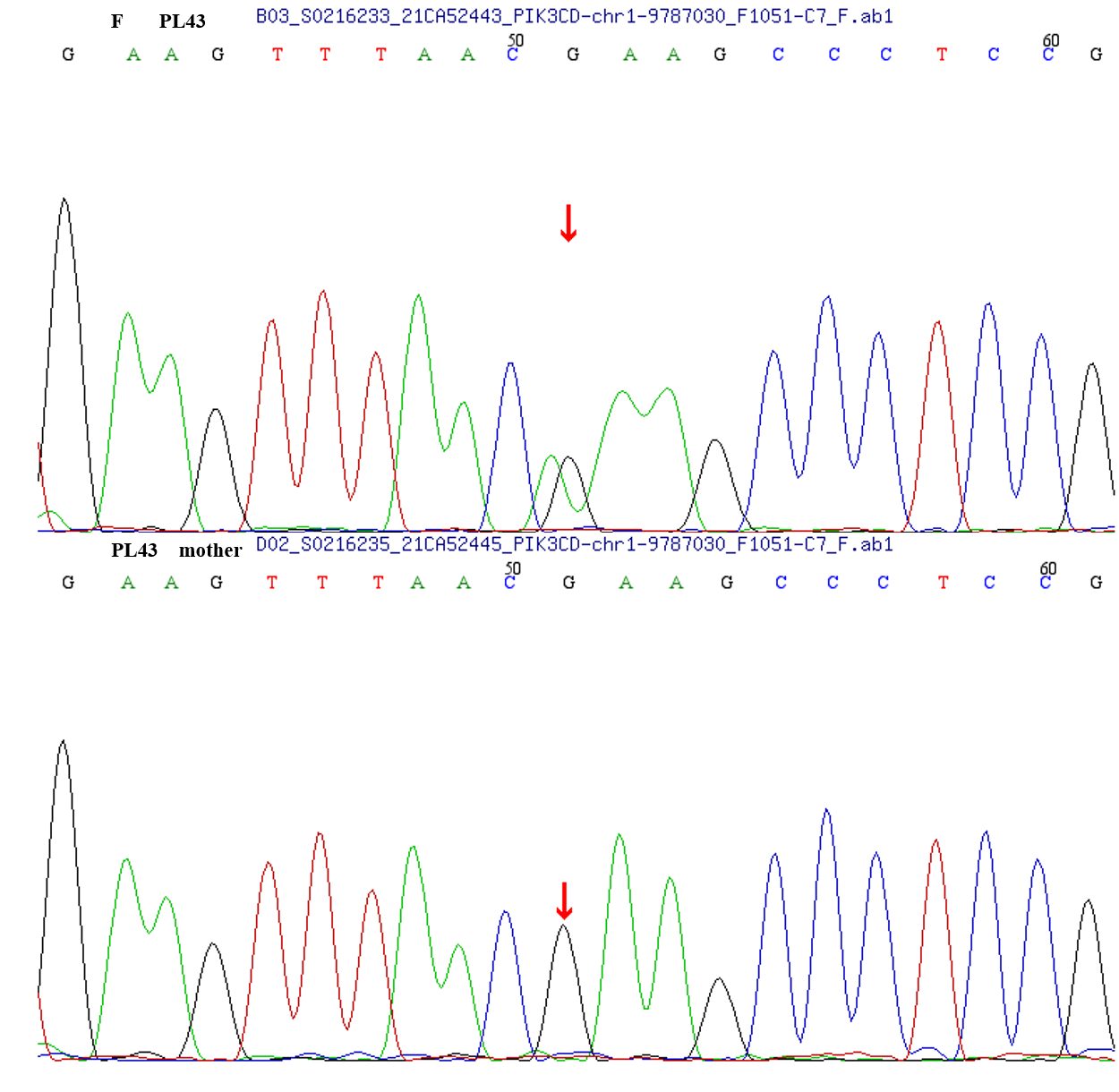

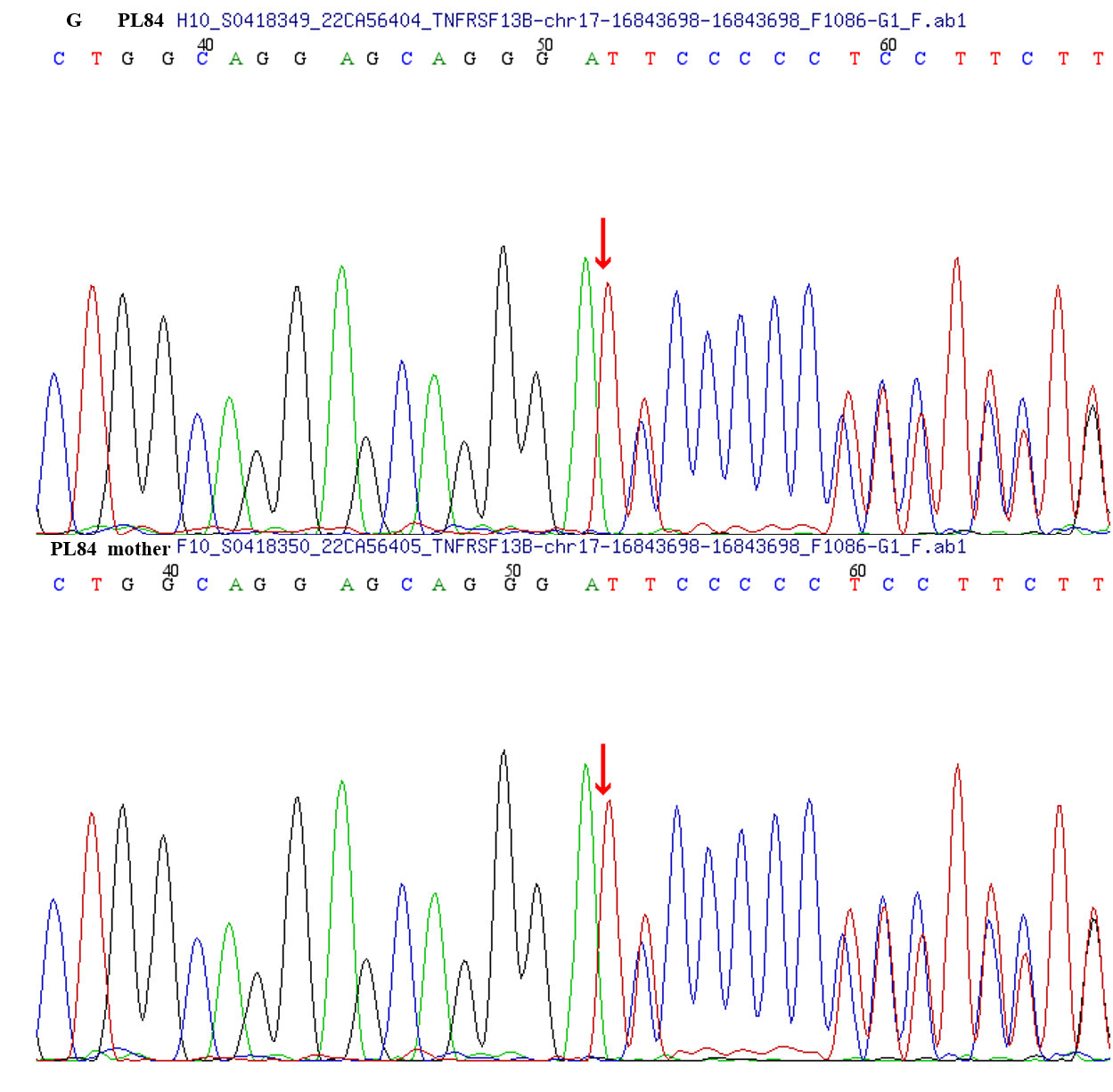

Supplement: Supplementary file 1 [file mmc1.docx]
